# Supplementary material for: The development of Vi-Connect: An educational game for the social inclusion at school of students with vision impairment
Source: PLoS One. 2024 Dec 5;19(12):e0306805. doi: 10.1371/journal.pone.0306805 (PMC11620618; doi:10.1371/journal.pone.0306805)
Supplement: Attachment — Submitted filename: Response to Reviewers.docx [file pone.0306805.s001.docx]

**Response to the Journal and Reviewers**

**Journal Requirements**

When submitting your revision, we need you to address these additional requirements.

**1. Please ensure that your manuscript meets PLOS ONE's style requirements, including those for file naming. The PLOS ONE style templates can be found at** 
[**https://journals.plos.org/plosone/s/file?id=wjVg/PLOSOne_formatting_sample_main_body.pdf**](https://journals.plos.org/plosone/s/file?id=wjVg/PLOSOne_formatting_sample_main_body.pdf) **and** 
[**https://journals.plos.org/plosone/s/file?id=ba62/PLOSOne_formatting_sample_title_authors_affiliations.pdf**](https://journals.plos.org/plosone/s/file?id=ba62/PLOSOne_formatting_sample_title_authors_affiliations.pdf)

Thank you for this very useful comment. We have now made sure that our manuscript meets PLOS ONE's style requirements, including those for file naming.

**2. Please note that funding information should not appear in any section or other areas of your manuscript. We will only publish funding information present in the Funding Statement section of the online submission form. Please remove any funding-related text from the manuscript.**

Thank you for this comment. No funding information is included in the manuscript. The following sentence included in the Acknowledgments section refers to Eye-YPAG and we have been advised to include it in this section as it is their standard practice when participating in peer-reviewed publications.

**3. Thank you for stating the following financial disclosure:  "****This research has been supported by the Public Engagement Fund from the University of Birmingham."**

**Please** **state what role the funders took in the study.  If the funders had no role, please state: "The funders had no role in study design, data collection and analysis, decision to publish, or preparation of the manuscript." 
If this statement is not correct you must amend it as needed. 
Please include this amended Role of Funder statement in your cover letter; we will change the online submission form on your behalf.**

Thank you for this useful suggestion. This information has been now included in our cover letter.

**4. In this instance it seems there may be acceptable restrictions in place that prevent the public sharing of your minimal data. However, in line with our goal of ensuring long-term data availability to all interested researchers, PLOS’ Data Policy states that authors cannot be the sole named individuals responsible for ensuring data access (http://journals.plos.org/plosone/s/data-availability#loc-acceptable-data-sharing-methods).**

**Data requests to a non-author institutional point of contact, such as a data access or ethics committee, helps guarantee long term stability and availability of data. Providing interested researchers with a durable point of contact ensures data will be accessible even if an author changes email addresses, institutions, or becomes unavailable to answer requests.**

**Before we proceed with your manuscript, please also provide non-author contact information (phone/email/hyperlink) for a data access committee, ethics committee, or other institutional body to which data requests may be sent. If no institutional body is available to respond to requests for your minimal data, please consider if there any institutional representatives who did not collaborate in the study, and are not listed as authors on the manuscript, who would be able to hold the data and respond to external requests for data access? If so, please provide their contact information (i.e., email address). Please also provide details on how you will ensure persistent or long-term data storage and availability.**

Thank you for this comment. As is usual for qualitative data we are not ethically permitted to share the full dataset including transcribed conversations with participants. In the study information sheets provided to participants we stated that all the information they provided will be stored on a local password-protected server accessible only to members of the research team. As such we are unable to share the data widely.

**5. When completing the data availability statement of the submission form, you indicated that you will make your data available on acceptance. We strongly recommend all authors decide on a data sharing plan before acceptance, as the process can be lengthy and hold up publication timelines. Please note that, though access restrictions are acceptable now, your entire data will need to be made freely accessible if your manuscript is accepted for publication. This policy applies to all data except where public deposition would breach compliance with the protocol approved by your research ethics board. If you are unable to adhere to our open data policy, please kindly revise your statement to explain your reasoning and we will seek the editor's input on an exemption. Please be assured that, once you have provided your new statement, the assessment of your exemption will not hold up the peer review process.**

As previously mentioned, it has been clarified in the study information sheets that only members of the research team will have access to the data. In line with the ethics guidelines of the University of Birmingham and the ethics approval of the study, recordings and transcripts of this study cannot be shared with the public. During our focus groups with participants, very sensitive topics regarding the mental health of a very vulnerable student population were discussed. Further, considering the very specific population that this study focused on, our data can be highly identifiable.

**6. Please review your reference list to ensure that it is complete and correct. If you have cited papers that have been retracted, please include the rationale for doing so in the manuscript text or remove these references and replace them with relevant current references. Any changes to the reference list should be mentioned in the rebuttal letter that accompanies your revised manuscript. If you need to cite a retracted article, indicate the article’s retracted status in the References list and also include a citation and full reference for the retraction notice.**

Thank you for the helpful comment and suggestion. We have now checked and updated our reference list.

**Response to Reviewers’ Comments**

We would like to thank very much the reviewers for their very helpful comments and suggestions. Please read our responses below.

***Reviewer #1: Review Report***
**Dear authors, thank you for submitting your manuscript to our journal. While it is a commendable piece of work, we offer a series of comments to enhance the quality of your manuscript.
Manuscript: "The Development of Vi-Connect: An Educational Game for the Social Inclusion at School of Students With Vision Impairment"**
Thank you very much for all your comments and suggestions. Please find our responses below.

**Theoretical Framework:
Suggested Improvement: Expand the theoretical review to include comparative studies of similar tools in populations with other disabilities, to contextualize the uniqueness and added value of Vi-Connect.**

Thank you for your comments. Based on your suggestions, more studies and additional information have been added to paragraphs 3 and 4 of the Introduction. In particular, the following paragraphs have been added to the Introduction:

**Paragraph 3:** *Although this is the first study that aims to develop a serious game for the school inclusion of young people with vision impairment, previous literature underscores the positive role of digital interventions in the form of educational games on social competence (12), self-esteem development and communication (13), and emotional regulation (14). Digital games have been utilised as primary and secondary interventions across natural settings such as schools and home showing promising outcomes in the social and cognitive abilities of students with neurodevelopmental and SEND such as dyslexia, Autism Spectrum Disorder (ASD), and ADHD (15). A review of serious games for autistic children has underscored the positive effect of such interventions on the expression of emotions and social engagement (16). More research on the design and development of serious games for autistic children has shown their positive impact on the development of social skills and interactions with their typically developing peers (17). Further, a research study focusing on the design of a serious game aimed at improving the independent living skills of people with intellectual disability and ASD highlighted the benefits of co-produced learning interventions in the forms of serious games for people with SEND (18). Although the focus of this prior study was not on vision impairment, the design framework of the aforementioned intervention which is based on the elements of participatory research, self-learning and personalisation can be particularly useful for future studies aimed at designing and developing serious games for students with SEND. The findings of a meta-analytic review showed that serious games have the potential to advance knowledge, reduce mental health difficulties and find applications with diverse groups (19). Another systematic review suggested that serious games can enhance social skills in classroom settings supporting students to practice and master social skills in low-risk environments (20).*

**Paragraph 4:** *A recent study by Giannakopoulos et al. (2018) aimed at developing a series of serious and inclusive games for children and young people with vision impairment aged 7-10 years showed the positive effects of participatory research and co-production with people with lived experience and professionals in the development and evaluation of such interventions (23).*

**Update of Sources:
Suggested Improvement: Include more recent studies on the impacts of the pandemic on the education of students with visual impairments, strengthening the justification of the study in the current context.**

Thank you so much for this very helpful comment. Based on your suggestions, we have added a paragraph focusing on the effect of COVID-19 on the social emotional development of people with vision impairment and on the mental health of children and adolescents with Special Educational Needs and Disabilities. As mentioned in this paragraph, there is an apparent lack of studies focusing on the impact of COVID-19 on the social emotional development and inclusion of visually impaired children and youth. However, it is expected that the aforementioned findings will also apply to the visually impaired student population who were also sensitive to the significant impact of COVID-19 on educational design and delivery.

Based on the reviewer’s suggestions, the following paragraph has been added to the Introduction:

*Recent research suggests that COVID-19 also had a significant negative effect on the mental health (7) and social interactions of people with vision impairment (8), especially the population with severe sight impairment (previously “blindness” according to the UK classification system). A study by Heinze et al. (2021) focusing on the long-term effects of COVID-19 on adults with vision impairment showed increased levels of loneliness compared to the typically developing population (9). The limited research of the impact of COVID-19 on the mental health of children and adolescents with Special Educational Needs and Disabilities (SEND) has associated lockdowns and distance learning with an increased number of psychological threats (e.g., social isolation and emotional neglect) and reduced coping mechanisms in these students (10). Previous research has also highlighted the negative effects of COVID-19 on the general anxiety levels and adaptive behaviours of children and adolescents with SEND with many participants displaying maladaptive behaviours (11). Thus, it is expected that the above findings will also apply to the student population with vision impairment who were also susceptible to the significant effect of COVID-19 on educational design and delivery. Considering the above findings that have highlighted the detrimental effect of COVID-19 on the social emotional development of individuals with vision impairment, there is an urgent need to address these challenges and provide appropriate educational support tailored to the specific needs of people with vision impairment.*

**Appropriateness of Objectives:
Suggested Improvement: Refine the objectives to include specific goals related to the socio-emotional skills that Vi-Connect aims to develop, providing clarity in the game's expectations.**

Thank you for this comment. Based on your comments and the comments of the second reviewer, additional information has been added to the first two paragraphs of the “The Current Study” subsection to provide clarity on the expectations of the game:

*This study aimed to initiate the development of an accessible educational game to address the social emotional, communication and self-advocacy needs of adolescents aged 12-16 years with vision impairment prioritising social relationships with teachers and classmates in school, in line with the recommendations of Area 9 “Health: Social, Emotional, Mental and Physical Wellbeing” of the Curriculum Framework for Children and Young People with Vision Impairment (CFVI). The socio-ecological model that has been developed by the first author of the study (Manitsa, 2023; Manitsa et al., 2023) based on Bronfenbrenner’s ecological systems theory (26) has informed elements of Vi-Connect, which emphasises school belonging as well as peer and teacher support as significant social factors for the social emotional development of adolescents with vision impairment (3). The proposed socio-ecological model provides a conceptual framework to understand and promote the social inclusion of students with vision impairment in school with a specific focus on the social (social relationships and participation in school activities), cultural (country of residence and education system) and chronological (age of the individuals) factors that may affect school belonging. Our previous research also highlighted the importance positive social relationships with peers and staff in the school belonging of adolescents with vision impairment (27). Therefore, the main purpose of Vi-Connect is to promote the school-based social relationships of students with vision impairment through the development of social skills that can have a positive impact on such relationships.*

*A range of social skills were targeted in this study based on the CFVI and previous literature on social emotional challenges experienced by adolescents with vision impairment in school (see (24,25,28–32). Based on the CFVI and previously reviewed literature focusing on the social challenges faced by students with vision impairment, the specific social skills that Vi-Connect targeted are the following: self-advocacy, problem solving skills, contact initiation, making and maintaining relationships, self-efficacy and agency, and confidence to interact with others independently. Three scenarios were developed that aimed to improve these social skills by promoting social relationships with teachers and peers. Similar to the design framework of other studies focusing on the design and development of educational games for students with SEND (18,23), the main purpose of the study was to initiate and promote self-learning, personalisation and continuous challenge.*

Further, additional information has been added to the “Phase 2: Development of the Prototype” section to remind the reader of the exact social skills that Vi-Connect aims to develop:

*Specifically, as discussed in the Introduction, Vi-Connect targets the following social skills: self-advocacy, problem solving skills, contact initiation, making and maintaining relationships, self-efficacy and agency, and confidence to interact with others independently.*

**Methodology:
Suggested Improvement: Detail the participant selection process and the inclusion/exclusion criteria to strengthen the methodological validity.**

Thank you for this comment. Additional information has been added to the last paragraph of the Introduction section (“The Current Study” subsection) and to the first two paragraphs of the Participants subsection.

**Introduction:** *Finally, people with vision impairment in the UK are categorised into two groups according to their registration status: those with severe sight impairment (previously “blindness” according to the UK classification system) and those with sight impairment (previously “low vision” according to the UK classification system). Considering the additional adaptations that students with severe sight impairment may require, the focus of this study was on students with sight impairment only. Further, students with intellectual disabilities and complex needs did not participate in this study due to the potential impact of these additional needs on their participation in an online focus group and in the implementation of the educational intervention. Taking into account their diverse needs and different types of social interactions that occur in specialist educational settings, students from special schools did not participate in this study. Also considering that current evidence for educational interventions targeting the social emotional support provided to students with vision impairments is mainly drawn from research on younger children (33) and considering the challenges specific to adolescence discussed above, the focus of this study was to develop an intervention with and for adolescents with vision impairment currently attending mainstream schools only.*

**Participants subsection:** *All participants were based in the UK at the time of the study. All adults participating in the study were required to have professional experience working with adolescents with vision impairment. No specific years of experience with adolescents with vision impairment were required for the study. Some professionals also had lived experience of vision impairment (that of a close relative).*

*All adolescents in the study had to be registered as sight impaired (previously “with low vision” according to the UK classification system), aged 12-16 years and attending mainstream schools. Adolescents with additional needs and from special schools did not participate in this study. Participants for the youth advisory groups fell into two categories: an online group and an in-person group.*

**Ethical Aspects:
Suggested Improvement: Describe the measures taken to ensure the accessibility and usability of the game for participants with visual impairments, ensuring equity in participation.**

Thank you for this comment and suggestion. As discussed in the Materials subsection, in the first phase of the study participants (adolescents with vision impairment and professionals) were asked to comment on specific accessibility features they find useful and game platforms they find accessible when playing video games. In the assessments phase of the study, all participants were again asked to comment on the accessibility features of Vi-Connect and offer suggestions for improvement. In the Levels and Aesthetics subsection of the Results section, we also explain specific accessibility features that have been included in Vi-Connect based on participant feedback*: “Considering feedback from adolescents and professionals, the levels use high contrast background (black background whenever possible) and adjustable font size (see Fig 5) to ease the distinction of different elements on the screen.”.* Similar information has been added to the Mechanics and UI subsection: *“Following suggestions from Phase 1 participants and to promote player understanding of the game, each line of dialogue plays an audio transcript when the text appears. Based on the participants' suggestions, the user can increase or decrease the font size of the text displayed in the dialog boxes.”*. Some information focusing on accessibility features can also be found at the end of the third paragraph of the Discussion: *“Regarding the accessibility features included in Vi-Connect, according to participant feedback, high quality audio, clear voices, simple graphics (e.g., black background) and reduced background noise have been included in the game.”.* As mentioned in the participants sub-section and the discussion, the focus of the current game was adolescents with sight impairment meaning that the players had some residual vision. Therefore, more adaptations are required for student populations with more severe sight impairment.

**Discussion:
Suggested Improvement: Deepen the discussion on how Vi-Connect aligns with the principles of inclusive education and social learning theory.**

Thank you for this very helpful suggestion. The second paragraph of the discussion is now focusing on the principles of inclusive education and social learning theory:

*Vi-Connect builds on the CFVI principles and areas suggested Douglas et al. (2019) (38). of fostering autonomy and agency in this population with digital technology by targeting the learning areas proposed: independence skills, social emotional functioning, and school inclusion. Our proposed digital intervention is also in line with the basic principles of inclusive education and social learning theory. In particular, the key principle of inclusive education is that all students should be included in the same classrooms and learn with and from each other despite their different needs and abilities (39). Considering the key proposals of inclusive education, the main goal of the suggested intervention is to improve the social interactions and relationships that adolescents with vision impairment develop with their teachers and classmates in mainstream schools and promote their social inclusion. In addition, through observing of the Main Character of Vi-Connect and the impact their choices can have on their school life, players with vision impairment are expected to learn how to make beneficial choices that promote their wellbeing and school inclusion. The self-learning component of Vi-Connect derives its key features from Bandura’s social learning theory, which proposes that observation and imitation promote the development of prosocial behaviours (40).*

**Suggested Improvement: Analyze the implications of the findings for developers of digital educational tools, offering recommendations based on the study's experience.**

Thank you for this great suggestion. More details on the implications of the findings for developers of digital educational tools has been added to the fifth paragraph of the Discussion section:

*Such findings may be particularly useful to other researchers and developers of educational games for students with vision impairment, as they may offer insights into the lived experiences of these students and the social elements that should be prioritised in such games. The adolescents who participated in this study reflected on their school experiences and reported the social emotional challenges they face at school. Such challenges could potentially be the main focus of future digital interventions aimed at promoting the school inclusion of children and young people with vision impairment. Our participants also referred to key features of the game that promoted the accessibility of Vi-Connect (e.g., audio, graphics, and font size). Such accessibility features can be particularly useful to other game developers who design educational games for people with vision impairment. The co-production approach we took to developing Vi-Connect highlights the importance of involving people with lived experience in developing such games.*

**Conclusions:
Suggested Improvement: Clarify how Vi-Connect addresses the deficiencies identified in the theoretical framework, directly linking the study's results with the stated objectives.**

Thank you for your comment. In the last paragraph of the Discussion, we added more details which link the gaps of previous literature to the development of Vi-Connect. In particular, we mentioned that the majority of previous studies focus on current challenges rather than solutions and are also targeting younger children rather than adolescents with vision impairment. We also provided literature which underscores the lack of educational interventions that can be implemented at home and the limited mental health support offered to children with SEND and people with vision impairment.

**Suggested Improvement: Include reflections on the scalability and sustainability of Vi-Connect in different educational settings.**

Thank you for your comment and suggestions. Also taking into account your subsequent comments focusing on study limitations, we have expanded the paragraph focusing on study limitations and suggestions for future research and also explained that the current prototype is not designed for special schools. We hope the current information pertains to your comment and is sufficient.

**Limitations and Suggestions for Future Research:** *This is one of the first studies that involved students with vision impairment in intervention development and planning. Despite the transformational effects of co-production and participatory research to service development (43) evident in the current study that prioritised the lived experiences of adolescents and professionals in the field of vision impairment, there are limitations that should be further discussed and suggestions for future research. First, Vi-Connect in its current form consists of three stories that focus on the social relationships that students with vision impairment develop at school, therefore other relevant scenarios and challenges that arise in the school environment are not included in this prototype. Second, some participant suggestions and feedback from Phase 3 were not embedded into the current version of Vi-Connect. For example, adolescents mentioned that their teachers should also play the game as this would help them better understand their needs. Previous research has shown that implementing disability awareness interventions among non-disabled individuals can increase tolerance and acceptance of people with disabilities and diverse needs (44). This suggestion could potentially be incorporated into a multiplayer version of Vi-Connect where students with vision impairment would be invited to play alongside their sighted peers. Multiplayer educational games have been found to have more positive effects on learning outcomes compared to single-player games (45). Third, as previously discussed, the focus of the current game was adolescents with sight impairment meaning that the players had some residual vision. Thus, more adaptations are required for student populations with more severe types of sight impairment. Also considering that adolescents with intellectual disabilities and complex needs were not involved in the development and assessment of Vi-Connect, the current prototype has not been designed for special schools. From our findings, it is evident that future research should prioritise adapting and customising Vi-Connect to meet the diverse needs of students with vision impairment and to be applicable in different educational contexts. Fourth, the current study focused on a very specific student population with vision impairment, including five students aged 12-16 who were registered as sight impaired (or “with low vision”). Furthermore, only a limited number of professionals participated in the study and all our participants were based in the UK at the time of the interview. Considering, therefore, the small size of the study and cultural differences that might have significantly influenced our findings (31), it would be useful to conduct cross-cultural studies with larger samples sizes and more diverse populations in terms of age and special needs. It would also be useful to conduct longitudinal studies to assess the effectiveness of Vi-Connect over time and its long-term impact on the social inclusion of students with vision impairment.*

**Limitations:
Suggested Improvement: Explicitly acknowledge the limitations related to the study design, such as sample size and geographical scope, and their impact on the generalization of the results.**

Thank you for your comment and suggestions. As previously mentioned, we have expanded the paragraph focusing on study limitations and also addressed the limited sample size and geographical scope of the study. We have also offered suggestions for future research based on your very helpful comments.

**Future Prospects:
Suggested Improvement: Propose** **longitudinal studies to evaluate the effectiveness of Vi-Connect over time and its long-term impact on the social inclusion of students.**

Thank you. We have now added this suggestion for future research at the end of the limitations subsection (paragraph added above).

**Suggested Improvement: Suggest** **future research focused on adapting and customizing the game to meet diverse needs and educational contexts.**

Thank you for this very useful suggestion. We believe that this comment links to your previous comment on the scalability and sustainability of Vi-Connect in different educational settings. Therefore, additional information has been added after this limitation in order to discuss ways to move forward and suggestions for future research.

**General Conclusion:
The manuscript presents an innovative and relevant study with the potential to positively impact the inclusion of students with visual impairments. To strengthen the study's contribution, we recommend an expansion and deepening in certain methodological, theoretical, and analytical aspects, as well as a more detailed discussion on the practical implications of the findings and future research directions.**

We would like to thank you for all your comments and constructive feedback that greatly improved our manuscript. We hope we have sufficiently responded to your comments.

**Reviewer #2**

**This is a valuable and worthwhile piece of research that has truly involved young people with vision impairment. It is good to note that there has been the genuine involvement of Eye-YPAG as well as other participants as part of the development of a digital intervention to support the social inclusion of students with vision impairment.
The authors have provided a strong rationale for this research that is clear and valid.
The methods are clearly outlined and the ethical considerations are thorough. The procedures are technically sound and the research enables the team to achieve their aims. This research is very specific and the conclusions are supported by the data gathered.** **Just a few points to consider:**
**Where Bronfenbrenner’s ecological systems theory is mentioned (line 95) it would be helpful to explain how this theory has informed elements of Vi-Connect. This is not clarified later in the description of the game.**

Thank you for this very helpful comment and suggestion. Based on your comments and the comments of the first reviewer, additional information has been added to the last two paragraphs of the Introduction and hopefully provides more clarity on how Bronfenbrenner’s ecological systems theory has informed elements of Vi-Connect.

**The presentation of the themes and the selected quotes from the participants around their social experiences within school provide important data (aside from the development of a digital intervention tool).**

Thank you very much for your positive comments. **The construction of the game is outlined clearly and the scenarios are well-described. The participants have been enabled to respond to the game and suggest improvements.**

Thank you for your positive comments. **Page 44 (line 51) There is a full stop missing after (3):
Adolescents with vision impairment experience significant barriers in school in relation to social acceptance, social adaptation, academic performance, peer relationships and self-esteem, mutually affecting their sense of belonging (3).**

Thank you for this comment. We have now corrected this typographical error. **Line 52: There is a full stop missing after (4):
They also struggle to fit in the wider school community often 52 resulting in smaller peer networks or isolation (4)**

Thank you for this comment. We have now corrected this typographical error. **Line 61: There is a full stop missing after (10):
Serious games have been utilised as primary and secondary 59 interventions across natural settings such as schools and home showing promising outcomes in 60 neurodevelopmental and Special Educational Needs and Disabilities (SEND) such as dyslexia, 61 autism, and ADHD (10)**
Thank you for this comment. We have now corrected this typographical error.

**Line 90: Please would it be possible to reference this: Curriculum Framework for Children and Young People with Vision Impairment (CFVI) (i.e. Hewett, R., Douglas, G., McLinden, M., James, L., Brydon, G., Chattaway, T., Cobb, R., Keil, S., Raisanen, S., Sutherland, C., Taylor, J. (2022). Curriculum Framework for Children and Young People with Vision Impairment (CFVI): Defining specialist skills development and best practice support to promote equity, inclusion and personal agency. RNIB)**
Thank you for your comment. The aforementioned reference has now been added to our reference list.

**Line 194: Typo: Tour research assistants then analysed the transcriptions 195 independently (Tour should read as Four).**

Thank you for this comment. We have now corrected this typographical error. **Line 425: GCSE’s – no apostrophe required.**

Thank you for this comment. We have now corrected this typographical error. **Line 499: CFVI principles. Reference Hewett et al. (2022) as above.**

Thank you for your comment. The aforementioned reference has now been added to our reference list. **Line 533: participatory research to service development (30) evident – space missing**

Thank you for this comment. We have now corrected this typographical error.

**Line 683: Inconsistent referencing. This should be Douglas, G., McLinden, M etc**

Thank you for your comment. This reference has now been updated.
