# Supplementary material for: The development of Vi-Connect: An educational game for the social inclusion at school of students with vision impairment
Source: PLoS One. 2024 Dec 5;19(12):e0306805. doi: 10.1371/journal.pone.0306805 (PMC11620618; doi:10.1371/journal.pone.0306805)
Supplement: Attachment — Submitted filename: Response to Reviewers.docx [file pone.0306805.s002.docx]

**Response to the Journal and Reviewers**

**Journal Requirements**

**Please review your reference list to ensure that it is complete and correct. If you have cited papers that have been retracted, please include the rationale for doing so in the manuscript text, or remove these references and replace them with relevant current references. Any changes to the reference list should be mentioned in the rebuttal letter that accompanies your revised manuscript. If you need to cite a retracted article, indicate the article’s retracted status in the References list and also include a citation and full reference for the retraction notice.**

Thank you for the helpful comment and suggestion. We have now checked and updated our reference list.

**Response to Reviewers’ Comments**

We would like to thank very much the reviewers for their very helpful comments and suggestions. Please read our responses below.

**Reviewer #1: Manuscript PONE-D-24-06103R1**

**Dear authors,**

**I have reviewed the revisions made by the authors in response to the reviewers' comments for the manuscript titled "The Development of Vi-Connect: An Educational Game for the Social Inclusion at School of Students With Vision Impairment." Below is a detailed evaluation of the adequacy of the changes made and additional recommendations.**

**Evaluation of the Revisions Made**

**Theoretical Framework and Literature Review:**

**The authors have expanded the theoretical review to include comparative studies of similar tools in populations with other disabilities and added a paragraph on the impacts of the COVID-19 pandemic on the education of students with visual impairments. These changes provide a broader and stronger context for the research and reinforce the study's justification in the current context.**

Thank you very much for your positive feedback.

**Study Objectives:**

**The study objectives have been refined to include specific goals related to the socio-emotional skills that Vi-Connect aims to develop. This addition improves the clarity and precision of the game's expectations.**

Thank you very much for your positive feedback.

**Methodology:**

**The process for selecting participants and the inclusion/exclusion criteria have been detailed, enhancing the transparency and methodological validity of the study. Additionally, measures taken to ensure the accessibility and usability of the game for participants with visual impairments have been described, ensuring equity in participation.**

Thank you very much for your positive feedback.

**Game Development and Content:**

**The authors have added details about the game's accessibility features based on participant feedback, ensuring the game's design is suitable for users with limited vision. They have also described the main character's features and the challenging social situations included in the game, which enhances the game's relevance and potential impact.**

Thank you very much for your positive feedback.

**Results and Discussion:**

**The discussion has been expanded to align with the principles of inclusive education and social learning theory. The authors have provided practical implications and recommendations for developers of digital educational tools, enriching the interpretation of the results and offering a useful guide for future developments in the field.**

Thank you very much for your positive feedback.

**Conclusions and Future Research:**

**It has been clarified how Vi-Connect addresses the deficiencies identified in the theoretical framework, providing a clearer connection between the results and the study's objectives. The authors have explicitly acknowledged the limitations related to the study design, such as sample size and geographical scope, and have proposed longitudinal studies to evaluate Vi-Connect's effectiveness over time.**

Thank you very much for your positive feedback.

**Additional Recommendations**

**Despite the significant improvements made, I suggest the following minor modifications for further clarity and coherence.**

**Terminology Clarification:**

**Ensure that the terminology used to describe individuals with visual impairments is consistent throughout the manuscript. For example, use terms like "visual impairment" or "limited vision" uniformly.**

Thank you very much for your comment. We would like to clarify that the terms “vision impairment” and “visual impairment” are used interchangeably in the UK to describe to describe vision loss that cannot be corrected by glasses or contact lenses. However, the term “vision impairment” is mostly used by the VI community, therefore we decided to proceed with this terminology. Furthermore, according to the NHS classification system, people with vision impairment in the UK can be classified as either severely sight impaired (blind) or sight impaired (partially sighted or with low vision); please see here for more information [The criteria for certification | RNIB](https://www.rnib.org.uk/your-eyes/navigating-sight-loss/registering-as-sight-impaired/the-criteria-for-certification/). The students who participated in our two online focus groups (before and after the development of the prototype) were registered as sight impaired (partially sighted) at the time of the study, therefore we need to make this distinction in our manuscript as it may affect our findings and the development of the intervention. We have discussed this further in the limitation paragraph where we have previously added the following: “Third, as previously discussed, the focus of the current game was adolescents with sight impairment meaning that the players had some residual vision. Thus, more adaptations are required for student populations with more severe types of sight impairment.”

**Game Implementation Details:**

**Include more details on how the game will be implemented in real educational settings. This could involve information about the necessary training for teachers and technical support to ensure effective integration.**

Thank you for this very helpful comment. Additional information has been added to the last paragraph of the manuscript on page 36, which explains that the proposed intervention aims to promote inclusivity by being both user and family friendly: *“In order for teachers and other school staff (e.g., teaching assistants and special educators) to promote Vi-Connect in the classroom, they do not need specialised training but access to a Windows device and an Internet connection. Students who wish to engage with Vi-Connect also do not need to receive special training or have previous experience playing video games, as there are detailed instructions and support (e.g. Games Master figure)* *to guide and help them to complete the intervention and also boost their confidence. The proposed intervention aims to promote inclusivity by being both user and family friendly.”*

**Citations and References:**

**Review and ensure that all citations and references are accurate and complete. Some minor typographical errors and inconsistencies in references should be corrected to maintain the academic accuracy of the manuscript.**

Thank you for the helpful comment and suggestion. We have now checked and updated our reference list.

**Impact Evaluation:**

**Include a brief discussion on how the long-term impact of the game will be measured beyond the initial evaluations. This will help contextualize the game's effectiveness and sustainability in different educational settings.**

Thank you for this very helpful suggestion. Additional information has been added on page 35 to further explain how the long-impact of Vi-Connect can be tested in more diverse populations: *“**Our aim is to evaluate the effectiveness of Vi-Connect and trial it with different student groups with and without additional sensory impairments (e.g., hearing impairment and multi-sensory impairment or deafblindness) in different geographical locations and schools. To trial Vi-Connect with different populations and assess its long-term impact, our research team is considering a range of quantitative and qualitative research methodologies that can be tailored to the learning needs of each student population, such as interviews and focus groups, large surveys and observational research. We are interested in conducting longitudinal studies with diverse group of students examining the effect of such digital interventions on their social competence, school engagement and academic performance. The proposed research methods will help us understand whether students attending more well-resourced schools are more likely to have positive mental health and social outcomes compared to young people from deprived areas. Applying a range of different research methods may further promote mixed methods approaches to the development of educational interventions for students with SEND as well as open the research field to designing more effective interventions for students with sensory impairments who have been under-represented in previous research.”.*

**Conclusion**

**Overall, the authors have adequately addressed all the reviewers' comments and suggestions. The changes made have significantly improved the clarity, coherence, and methodological rigor of the manuscript. With the additional minor modifications recommended, I suggest that the revised manuscript be considered for publication in PLOS ONE.**

Thank you very much for all your very positive feedback and suggestions.

**Reviewer #2: Thank you for this resubmission. I have now had the opportunity to read through the revised manuscript. I am happy that the suggestions that I have made have been addressed.**

Thank you very much for all your very positive feedback and suggestions.
